# The Road toward a Trojan Horse Malaria Vaccine: Chemical Augmentation of Live Sporozoites without Affecting Their Viability

**DOI:** 10.1021/acs.molpharmaceut.6c00575

**Published:** 2026-08-23

**Authors:** Chanel Milène Naar, Roos van Schuijlenburg, Maarten van Meerbeek, Severine Chevalley-Maurel, Lili A. Zigó, Blandine Franke-Fayard, Fijs W. B. van Leeuwen, Meta Roestenberg

**Affiliations:** 1 Leiden University Center for Infectious Diseases (LUCID), Leiden University Medical Center, Leiden 2333ZA, The Netherlands; 2 Interventional Molecular Imaging Laboratory, Leiden University Medical Center, Leiden 2333ZA, The Netherlands

**Keywords:** *Plasmodium falciparum*, whole-sporozoite vaccine, genetically attenuated parasite, adjuvant−antigen complex, mitochondrial membrane potential loading, internalized drug loading

## Abstract

In our effort to design an effective ultralow-dose attenuated whole sporozoite (SPZ) vaccine, we explored the use of chemical adjuvants to increase the immunogenicity of sporozoites. Critical to the potency of attenuated sporozoites is their ability to migrate to and infect the liver. We thus used an *in vivo* mouse challenge model to compare the effects of external and internal chemical modifications to sporozoites. We loaded sporozoites with CL307 using two strategies: a supramolecular surface modification adjuvant system (SPZ-SAS­(CL307)) and a mitochondrial membrane potential system (SPZ-MMP­(CL307)). SPZ-SAS­(CL307) contained 200 times more CL307 as compared to SPZ-MMP­(CL307). However, SPZ-SAS­(CL307) could not establish a liver infection after intravenous injection (25 000 sporozoites), suggesting loss of function of the sporozoites. On the contrary, SPZ-MMP­(CL307) could establish an equivalent liver infection to the control already at a dose of 5000 sporozoites. When the immunized mice were challenged, SPZ-MMP­(CL307) equaled the protective efficacy of control sporozoites. Our findings indicate that the MMP system can be used to chemically augment live cells, such as sporozoites, with adjuvants without negatively affecting their infectivity and protective efficacy *in vivo*. For malaria, further testing of different types of adjuvants at different concentrations is needed to ultimately develop vaccines with increased potency. However, in essence, this platform provides a proof-of-concept for a Trojan horse adjuvanting or drug loading concept for any whole-cell vaccine or therapy, within the infectious diseases space or beyond in oncological applications.

## Graphical abstract

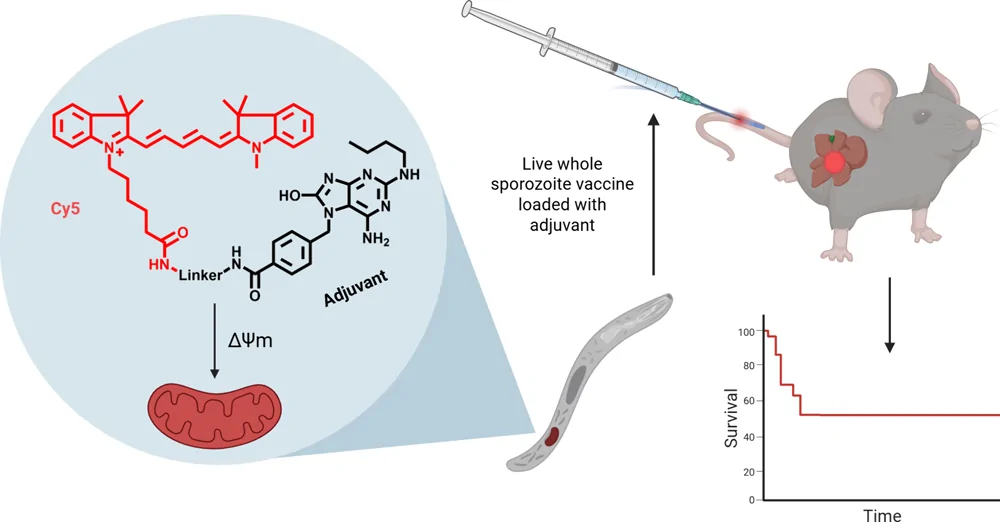

## Introduction

Live attenuated vaccines are traditional and powerful vaccines that make the cornerstone of disease control for many infectious diseases.^1^ Their success lies in the breadth of antigens that are exposed to the immune system in their natural context and the inherent immunogenicity of the attenuated pathogens. These vaccines have shown enormous global health impact and include vaccines for measles, mumps, rubella, yellow fever, polio, and rotavirus, among others.^1^ Similarly, live attenuated vaccines for malaria could help to prevent more than half a million annual deaths globally.^2^

Current live attenuated malaria vaccine candidates in human clinical trials use radiation attenuation or genetic modification of sporozoites (SPZ) that allows effective migration to the liver but prevent the onset of disease beyond the liver stage while retaining natural SPZ and liver stage immunogen exposure.^3–5^ In controlled human malaria infection models, we have shown that infectivity of whole sporozoite vaccines is critical.^3^ For example, a second generation of genetically attenuated sporozoites (GA2) that can still infect hepatocytes and show development up to late liver stage provide increased protective efficacy compared to an early arrestor.^3^ However, given the complexity of their production, using either laboratory-bred malaria-infected mosquitoes^6^ or *in vitro* culture production,^7^ is costly and scalability limited. Therefore, highly immunogenic, low doses are preferred. Hereby, it is crucial that such performance enhancements do not impair the SPZ viability or infectivity.

Due to long-term coevolution with the human host, SPZ have become inherently immune-evasive.^8^ To overcome this effect and enhance the SPZ immunogenicity, adjuvants can be used to orchestrate the type and magnitude of the immune response. However, the use of adjuvants also creates risks of systemic side effects, such as immunotoxicity and cytokine storms.^9^ A strategy used to reduce unwanted side effects is to bind the adjuvant to the antigen, ensuring that the adjuvant is only active where the antigens accumulate.^10^ Binding adjuvants to live SPZ, however, is much more challenging than the adjuvant conjugation to a protein or peptide.

There is growing interest across multiple disciplines in engineering cells as specialized drug delivery platforms. Cells are attractive carriers due to their biocompatibility, ability to traverse biological barriers, and inherent targeting capabilities.^11^ However, most current approaches rely on cell surface modifications, which can impair cell migration or homing by altering surface receptors and adhesion molecules.^12–14^ Intracellular loading could overcome this limitation but is technically challenging. Existing methods, such as viral transduction or membrane disruption, are unsuitable for the safe and scalable production of whole-cell therapies and vaccines,^15^ and robust alternative strategies have yet to be established.

Previously we functionalized the outer membrane of SPZ with CL307 using supramolecular chemistry (SPZ-SAS­(CL307)).^16^ This strategy demonstrated increased immune cell activation *in vitro* and significantly improved recruitment and activation of NK, CD4+, and CD8+ T cells to the liver *in vivo*,^16^ which not only aligned with the known effects of synthetic TLR7 agonists such as CL307 ^17^ but also linked with the known signatures of protection against liver stage malaria (e.g., IFNg-producing CD8+ T-cells.^18–20^ Unfortunately, the chemical functionalization of SPZ-SAS­(CL307) negatively impacted the SPZ motility and infectivity, which are critical parameters for vaccine potency. We thus proceeded to alternative strategies for TLR7 adjuvanting that preserve motility and infectivity. For this purpose, we developed an internal loading method using the SPZ’s mitochondrial membrane potential (MMP; SPZ-MMP­(CL307),^21^ a so-called “Trojan horse” strategy. This yielded significant improvements in immunological response *in vitro*, including macrophage activation and CD8+ T cell activation in a coculture.^21^ However, further insights into how this translates to protective efficacy *in vivo* are needed to iteratively improve this novel adjuvanting SPZ-vaccine concept and determine the next steps for optimization

In this study we evaluate how the “Trojan horse” MMP loading of SPZ with CL307 compares to the supramolecular adjuvant system (SPZ-SAS­(CL307)) *in vivo* in terms of viability, infectivity, and protective efficacy. We employed a GA2-like late-liver stage arresting *Plasmodium berghei* whole SPZ vaccine in a murine challenge model in which nonadjuvanted SPZ confer approximately 33% protection, providing a baseline for comparison. In doing so we pursued equivalence in terms of viability, infectivity, and protective efficacy to nonchemically modified SPZ and significant improvement over the supramolecular adjuvanted strategy, advancing a new path toward a future single-dose live chemically augmented malaria vaccine.

## Methods

### Production of Sporozoites

*Plasmodium berghei* sporozoites (SPZ) were obtained by infecting OF1 mice with an intraperitoneal injection of 10^5^ infected red blood cells. *Plasmodium berghei* late arresting genetically attenuated parasites (LA-GAP) PbΔ*mei2*Δ*lisp2* (2900cl3, mutant RMgm-4937; www.pberghei.eu) was used to generate SPZ for immunizations. This SPZ line is genetically attenuated by deleting the meiosis inhibited 2 (*mei2*) and liver-specific protein 2 (*lisp2*) genes, which ensures they arrest in late liver stage. For the challenge, the *P. berghei* ANKA line 1868cl1 was used (line RMgm-1320; www.pberghei.eu) which contains the reporter genes mCherry and luciferase under control of the constitutive *hsp70* and *eef1α* promoters, respectively, integrated into the neutral *230p* gene locus (PBANKA_0306000), providing a phenotype similar to wild-type.

Female *Anopheles stephensi* mosquitoes were allowed to feed when mice reached 0.2–0.8% blood gametocytemia as previously described.^22^ The *Pb*-infected mosquitoes were maintained at 21 °C at 80% relative humidity for 21–23 days. Thereafter, salivary glands of the infected and matched uninfected mosquito salivary gland extracts (SGE) were harvested via manual dissection. Salivary glands were homogenized to release SPZ in Roswell Park Memorial Institute medium (RPMI; Thermo Fisher Scientific). SPZ were then counted and used for experiments immediately.

### Experimental Animals

Four- to six-week-old female OF1 mice (SPZ production) and female C57BL/6J mice (immunization-challenge experiments, Charles River Laboratories) were acclimatized for 1 week prior to handling of them. Mice were housed with 3–5 mice per cage in ventilated cages with autoclaved aspen woodchip, fun tunnel, wood chew block and nestlets (12:12 h light–dark cycle; 21 ± 2 °C; relative humidity of 55 ± 10%). During the experiment, mice were fed with commercially prepared autoclaved dry rodent diet pellets and water, both available *ad libitum*. All animal experiments were granted with license 11600202216547 by the Competent Authority after advice on ethical evaluation by the Animal Experiments Committee Leiden and were performed in accordance with the Experiments on Animals Act (Wod, 2014), the applicable legislation in The Netherlands in accordance with the European guidelines (EU directive no 2010/63/EU). The study was executed in a licensed establishment for experimental animals.

OF1 mice were anesthetized at a parasitemia of 2% to 5% for mosquito feeding before malaria-associated symptoms occurred. Humane end points: the animals’ body condition was thoroughly examined daily. Animals were humanely sacrificed if the following defined end points were reached: visible pain (abnormal posture and/or movement), abnormal behavior (isolation, abnormal reaction to stimuli, no food and water intake). No mice were euthanized due to humane end points.

For the immunization-challenge experiments, we defined an end point cut off at 1% parasitaemia as counted by blood smear. Mice were checked for behavior and blood parasitemia every 24 h on days 4–14 postchallenge.

### Synthesis of Compounds

The supramolecular chemistry adjuvant system (SAS) compounds were synthesized as previously published.^16^ The Ad-Osu esters were prepared to contain adamantane and Cy3 to track the molecule. The host poly­(isobutylene-*alt*-maleic anhydride) (PIBMA389) polymers contained β-cyclodextrins, Cy5, and the adjuvant CL307.

The mitochondrial membrane potential (MMP) adjuvant compound was synthesized as described previously.^21^ The MMP­(CL307), in the referred article called Cy5-CL307, contains a Cy5 molecule directly linked to the adjuvant CL307.

### SPZ Adjuvanting

SPZ-SAS­(CL307) were generated as previously described.^23^ In short, the surface of SPZ was prefunctionalized with adamantane by incubating SPZ with 1 μM Ad-Osu ester and 10 μg/mL Hoechst 33342 in Roswell Park Memorial Institute medium (RPMI; Thermo Fisher Scientific) for 15 min at 37 °C. After washing twice with phosphate-buffered saline (PBS) supplemented with 1% fetal calf serum (FCS) through centrifugation at 13 000 relative centrifugal force (RCF) for 5 min, SPZ were resuspended in 1.9 × PBS with 1 μM CL307-bearing host polymer. SPZ were left to incubate for 4 h at 37 °C and mixed every half hour. Then, SPZ were washed thrice, resuspended, and counted.

SPZ-MMP­(CL307) were generated as previously described.^21^ In short, SPZ were incubated with 100 μM Cy5-CL307 in RPMI supplemented with 10% FCS for 15 min while shaking at 1000 rpm (RPM). Hereafter, they were washed thrice with RPMI with 10% FCS by centrifugation, resuspended, and counted using a Bürker counting chamber.

Lastly, a control was generated by using material from salivary glands of uninfected mosquitoes (SGE) and labeling it the same way with Cy5-CL307, resulting in SGE-MMP­(CL307).

### *In Vitro* Liver Infection Assay

A total of 40 000 HC-04.j7 hepatocytes (kindly provided by Dr. Rhoel Dinglasan) were seeded into NUNC 96-well optical glass-bottom plates (Thermo Fisher Scientific) precoated with 50 μg/mL collagen type I (Corning, USA) for 30 min at room temperature. Following overnight incubation at 37 °C with 5% CO_2_, the culture medium was replaced with 100 μL of DMEM containing 5% FCS, supplemented with 1% penicillin/streptomycin, 15 mM glucose, and 0.01 mg/mL insulin (all from Thermo Fisher Scientific, Waltham, MA, USA). For infection, sporozoites (SPZ) were added at a density of 60 000 per well in the same medium. Plates were centrifuged at 1200 rpm for 3 min and incubated for 24 h at 37 °C with 5% CO_2_. Cells were subsequently fixed with 3.7% paraformaldehyde (Sigma-Aldrich) for 15 min and permeabilized using 0.5% Triton (Thermo Fisher Scientific) for 20 min. Blocking was performed with 10% FCS in PBS prior to staining. Primary staining was carried out using HSP70 antibody (1:200; Thermo Fisher Scientific) overnight at 4 °C, followed by incubation with a secondary anti-rabbit AF594 antibody (1:500; Invitrogen) for 1 h. Nuclear staining was performed with Hoechst 33342 (1:2000; Sigma) for 30 min in the dark. PBS washes were performed between each step. Imaging was conducted using an ImageXpress confocal system (Molecular Devices), and HSP70-positive cells were quantified using MetaXpress high-content image acquisition and analysis software (Molecular Devices).

To determine if the adjuvant was present within infected hepatocytes, before the fixing of the cells, the plate was imaged using a Leica SP8 confocal fluorescence microscope (Leica Microsystems). Cy5 was excited using the white light laser at 633 nm, whereafter emission was captured at 650–700 nm.

### Immunization of Mice

One day prior to immunization, mice were randomized into groups of 9 mice (with 4 or 5 mice per cage). On the day of the immunizations, immunizations were prepared fresh by dissecting and adjuvanting SPZ just before use. SGE controls were generated from the same mosquito batch as the SPZ mosquitoes and contained the same number of mosquitoes as the matched SPZ immunization. The specific amounts of SPZ and thus matched SGE controls can be found in the figure legends. The labeling of the SPZ was checked by FACS to ensure 100% labeling was achieved. An independent person blinded immunizations to the study team until after data analysis. Immunizations were performed via intravenous injection in the tail vein after the mice are warmed under a heat lamp set at 35 °C. Second immunizations were always performed 7 days after the first with the same SPZ or SGE or RPMI dose.

### Quantification of Parasite Liver Load by *in Vivo* Bioluminescence Imaging

At 44 h postimmunization, the parasitic liver load was assessed using bioluminescent imaging as previously described.^24,25^ In short, mice were injected subcutaneously with d-luciferin (100 mg/kg in PBS; Caliper Life Sciences), and imaging was performed 8 min later using the IVIS Lumina II imaging system (PerkinElmer Life Sciences). Quantitative analysis of whole-body bioluminescence was conducted using Living Image 4.4 software by measuring signal intensity within defined regions of interest. Mice were not shaved as small wounds could affect the immune system and thus the reaction on the immunization.

The mice were checked for breakthrough infection at 5 or 6 days after immunization by blood smear.

### Challenge of Mice

The mice were challenged at 21 days after the (first) immunization with either 3000 or 30 000 *Pb* SPZ. Challenge SPZ were freshly prepared and administered via intravenous injection. The mice were then checked for blood stage parasites in a thin blood smear every 24 h from day 4 until day 14 postchallenge by taking a drop of blood from a tail cut. This blood smear was fixed with methanol and stained with Giemsa. Multiple fields of view were counted to reach at least 1000 red blood cells to calculate blood parasitemia. Mice were determined as blood-stage-positive at 1% blood parasitemia.

## Results

### SPZ Can Be Adjuvanted Using Different Chemical Strategies

Adjuvanted SPZ could effectively be obtained using both the SAS and the MMP platform, as both resulted in 100% labeling of SPZ (Figure 1A,B). For SPZ-SAS­(CL307), the adjuvant accumulated throughout the SPZ (Figure 1C), whereas the SPZ-MMP­(CL307) displayed focal mitochondrial accumulation (Figure 1D). Uniquely, in a subset of SPZ-MMP­(CL307), we observed whole SPZ cytosolic accumulation (Figure 1E). Over the whole population of adjuvanted SPZ, the SPZ-SAS­(CL307) formulation exhibited an 8.7 times higher median Cy5 fluorescence signal compared to SPZ-MMP­(CL307) (MFI 68498 vs 7886 respectively, Figure 1F,G). Given the known ratio of CL307 molecules per Cy5 unit in the polymer, this suggests that SPZ-SAS­(CL307) contains roughly 200 times more adjuvant per sporozoite than SPZ-MMP­(CL307).

**Figure 1:**
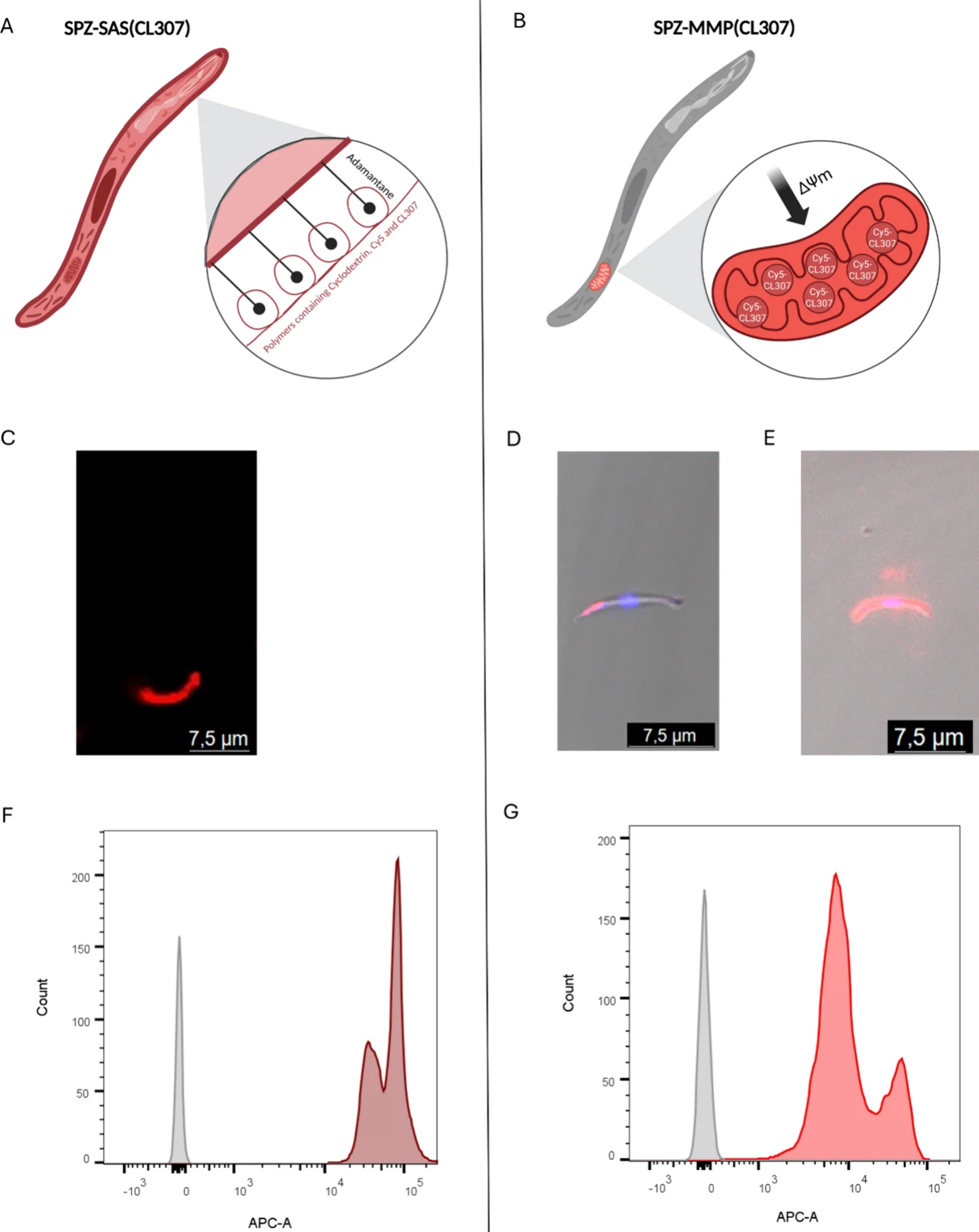
SPZ adjuvanting strategies: (A) Graphical description of the supramolecular adjuvant system (SPZ-SAS­(CL307)) adjuvanting procedure showing surface coating and cytosolic accumulation of adamantane compound, which links with polymers carrying CL307 and Cy5. (B) Graphical description of the mitochondrial membrane potential (SPZ-MMP­(CL307)) adjuvanting procedure showing mitochondrial uptake of Cy5-CL307 compound via the mitochondrial membrane potential. (C) Confocal microscopy image of SPZ-SAS­(CL307) with surface staining. Fluorescent image using the 633 laser to excite Cy5. (D) Confocal microscopy image of SPZ-MMP­(CL307) with mitochondrial staining. Fluorescent image using the 633 laser to excite Cy5, overlaid on a brightfield picture. Nucleus is stained with Hoechst in blue. (E) Confocal microscopy image of SPZ-MMP­(CL307) with cytosolic accumulation. Fluorescent image using the 633 laser to excite Cy5, overlaid on a brightfield picture. Nucleus is stained with Hoechst in blue. (F, G) Representative histogram of the fluorescence on the adjuvanted SPZ measured by flow cytometric analysis. Gray lines represent background signal in nonadjuvanted control SPZ. Dark red line shows Cy5 signal in the SPZ-SAS­(CL307) (F), and light red line shows Cy5 signal in the SPZ-MMP­(CL307) (G).

### Adjuvanted SPZ-MMP­(CL307) Remains Infective *in Vitro* and Cy5-CL307 Can Be Seen within the Hepatocytes

Using *in vitro* hepatocyte infection with SPZ and SPZ-MMP­(CL307), we observed that the SPZ-MMP­(CL307) remains infective (Figure 2A). However, the percentage of infected hepatocytes did decrease compared to the unadjuvanted SPZ (Figure 2A). When looking at these hepatocytes in detail with a confocal microscope, clusters of Cy5 could be observed in the SPZ-MMP­(CL307)-infected hepatocytes (Figure 2B). Previously, we have shown that the labeling of SPZ-MMP­(CL307) remains stable for at least 24 h.^21^ The SPZ-MMP­(CL307) thus remains infective and takes the compound into the hepatocytes with them.

**Figure 2:**
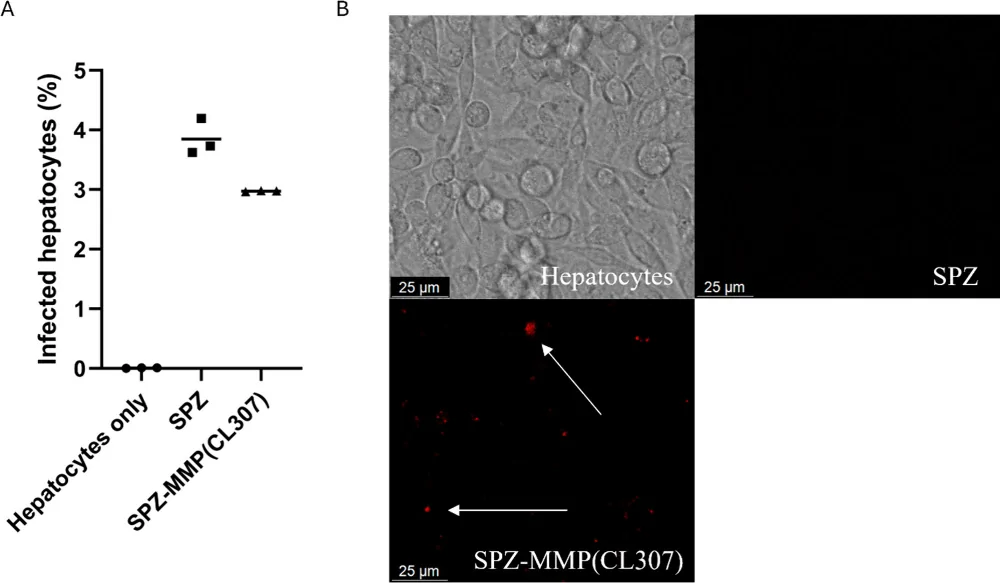
Hepatocyte infection of SPZ-MMP­(CL307) *in vitro*: (A) Percentage of infected hepatocytes for control SPZ and SPZ-MMP­(CL307). The wells were measured using the ImageXpress confocal (Molecular Devices), and the HSP70 positive cells were automatically counted by the high-content image acquisition and analysis software MetaXpress (Molecular Devices) to avoid bias. Data points are technical replicates. (B) Confocal image of live hepatocytes with clusters of Cy5 in SPZ-MMP­(CL307) infected hepatocytes. Only adjuvanted SPZ have the Cy5 label so clusters indicate SPZ that have infected a hepatocyte, while the SPZ panel serves as control to show no background autofluorescence was measured.

### Adjuvanted SPZ-MMP­(CL307) Remains Infective *in Vivo*

Bioluminescent imaging was used to evaluate the SPZ (control) (Figure 3A), SPZ-SAS­(CL307) (Figure 3B), and SPZ-MMP­(CL307) liver infectivity in mice. Following IV injection of 5000 sporozoites, the parasitic liver load was determined 44 h postinfection. In the 9 C57BL/6 mice analyzed per group, no significant difference in liver load could be observed between the unadjuvanted control SPZ and the SPZ-MMP­(CL307), with mean relative luminescence units (RLU) of 4.0 × 10^5^ and 2.3 × 10^5^, respectively (*p* = 0.131; Figure 3A,C). In contrast, no detectable liver load was seen in mice injected once with 25-fold more (125 000) SPZ-SAS­(CL307), clearly indicating a loss of infectivity (Figure 3B,C).

**Figure 3:**
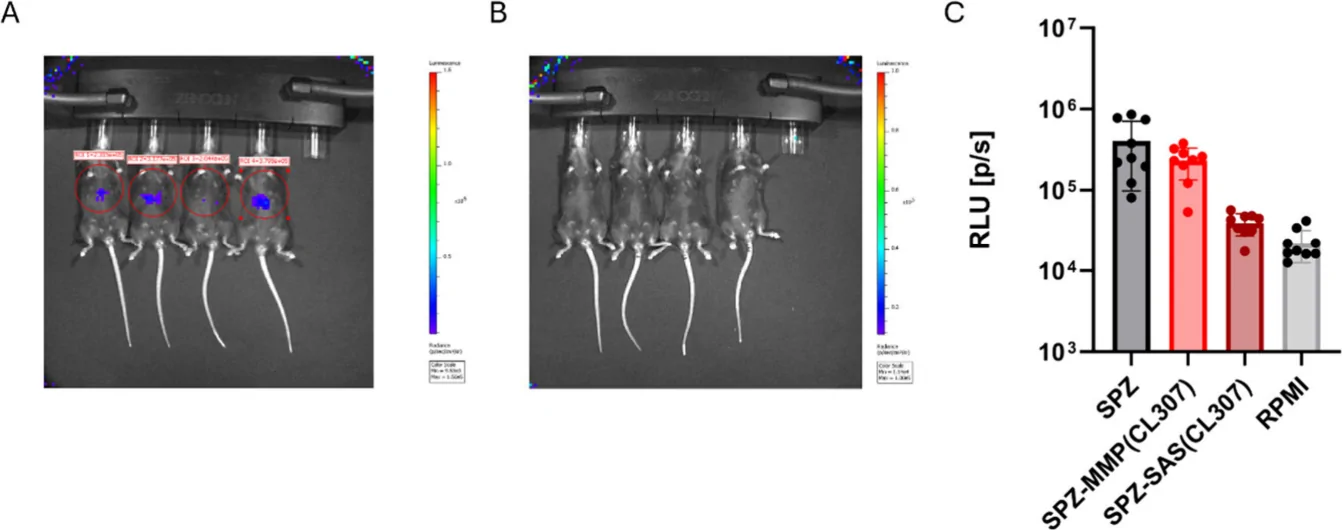
Liver load imaged with bioluminescence in *C57BL/6* mice: (A) Bioluminescence of SPZ-MMP­(CL307) after immunization as measured with the IVIS Lumina II imaging system. (B) Bioluminescence of SPZ-SAS­(CL307) after (first) immunization as measured with the IVIS Lumina II imaging system. (C) Quantification of liver loads in relative light units (RLU) with each dot representing a mouse and bars showing the mean and SD.

### Evaluation of the Protective Efficacy

To evaluate the protective efficacy, we set out to establish a challenge model that yielded partial protection with unadjuvanted control SPZ so that improvements in potency can be detected. However, we found that LA-GAP SPZ doses with both prime and boost immunizations (7 days apart) between 5000 and 25 000 all provided 100% protective efficacy (Figure 4A,B). Therefore, we proceeded with single-dose immunizations and identified that an ultralow dose of 5000 LA-GAP SPZ conferred a roughly 30% protection threshold (Figure 4A,B). Mice immunized with 5000 SPZ-MMP­(CL307) exhibited equivalent protection as unadjuvanted control SPZ despite chemical modifications, with 22% and 30% of mice protected, respectively (*p* = 0.383; Figure 4C). In contrast, SPZ-SAS­(CL307) failed to confer protection even with an intensified vaccination regimen that consisted of both prime–boost immunization and a 25-fold higher dose (125 000 LA-GAP SPZ), combined with a 10-fold reduced challenge dose (3000 SPZ, Figure 4C).

**Figure 4:**
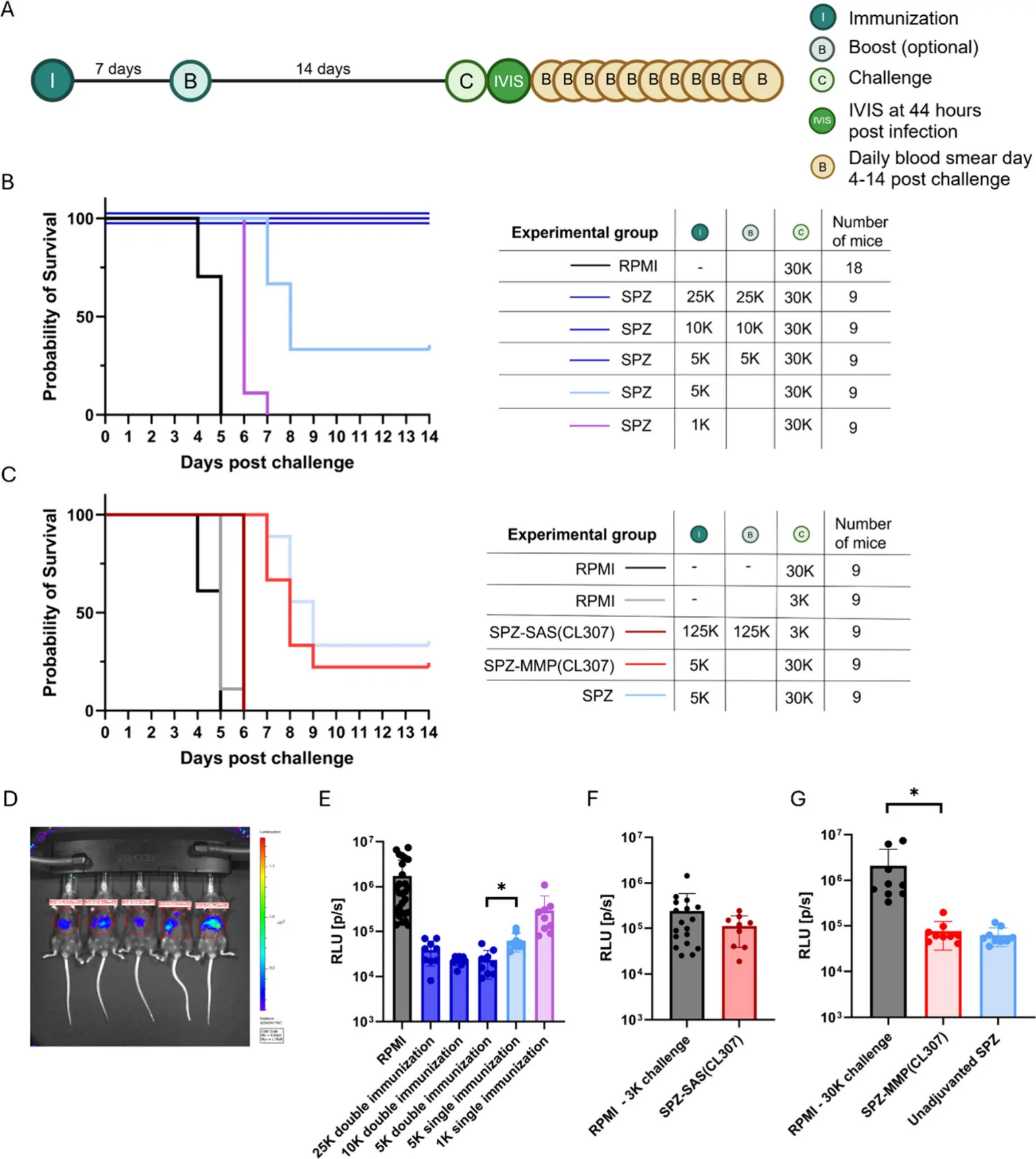
Similar protective efficacy of SPZ and SPZ-MMP­(CL307): (A) Schematic timeline of the mouse challenge model. Immunization (I), boost (B), and challenge (C) are shown as turquoise, light turquoise, and light green circles, respectively. Thereafter, the IVIS and daily blood smears are depicted in green and orange. (B) Setup of the model performed in 3 independent experiments: Kaplan–Meier survival curves of varying dosing unadjuvanted control groups consisting of RPMI control (black), double immunizations (dark blue), and 5000 (light blue) and 1000 (purple) single immunizations. Table including immunization (I), boost (B), and challenge (C) details of the varying unadjuvanted control dosing groups. Colored lines connect with the outcome of the different immunization experiments in the Kaplan–Meier curve. (C) Comparison of adjuvanted SPZ with control SPZ (1 independent experiment): Kaplan–Meier survival curves of adjuvanted SPZ groups showing RPMI controls challenged with 30 000 SPZ (black), RPMI controls challenged with 3000 SPZ (gray), SPZ-SAS­(CL307) (dark red), SPZ-MMP­(CL307) (light red), and control SPZ (light blue). Table including immunization (I), boost (B), and challenge (C) details of adjuvanted SPZ groups. Colored lines connect with the outcome of the different immunization experiments in the Kaplan–Meier curve. Control SPZ (light blue) and SPZ-MMP­(CL307) and RPMI-3K and SPZ-SAS­(CL307) are compared using the log-rank test (*p* = 0.383 and *p* < 0.001 respectively). (D) Liver load after challenge of RPMI group was imaged by bioluminescence using the IVIS Lumina II imaging system at 44 h after challenge. (E) Quantified liver loads in relative light units (RLU) for 3 different experiments using varying doses of unadjuvanted LA-GAP SPZ, e.g., 25K, 10K, and 5K double immunization (dark blue), 5K (light blue) and 1K (purple) single dose immunization with a high dose 30K challenge to establish the *in vivo* model. (F) Liver loads in RLU for individual mice in the SPZ-SAS­(CL307) (red) and RPMI group (black) after challenge with low dose 3K SPZ. Immunization dose was 125K for both groups. G) Liver loads in RLU for SPZ-MMP­(CL307) (red), RPMI (black), and unadjuvanted SPZ (light blue) groups after challenge with 30K SPZ. Immunization doses were 5K for all three groups. SPZ-MMP­(CL307) was compared to the RPMI or SPZ group using an independent samples Mann–Whitney *U* test. (*p ≤ 0.05).

### Postchallenge Liver Loads Significantly Reduced by SPZ-MMP­(CL307) Immunization

For a more sensitive measure of protection outcome after the challenge than time to blood-stage parasitemia, postchallenge parasite liver loads were quantified using *in vivo* imaging of the luciferase-expressing *Pb* parasites. Postchallenge liver load analysis reveals that reducing the immunization dose from double 5000 LA-GAP SPZ immunization to a single 5000 LA-GAP SPZ decreases protection against a high-dose (30 000) challenge from 100% to 30% (Figure 4B) while markedly increasing parasitic liver load after challenge (*p* = 0.002, Figure 4E). Further reducing the dose to 1000 LA-GAP SPZ results in an even higher liver load, demonstrating that differences in protection can be effectively quantified through liver load measurements in more detail and that the vaccine efficacy shows a dose-dependent trend (*p* = 0.06, Figure 4E). Comparing adjuvanted LA-GAP immunized groups revealed that postchallenge parasitic liver loads were comparable between SPZ-MMP­(CL307) immunized and unadjuvanted SPZ immunized groups at a dose of 5000, with mean RLU of 7.6 × 10^4^ and 6.3 × 10^4^, respectively (*p* = 0.462; Figure 4G). For the SPZ-SAS­(CL307)-immunized mice we observed no protected mice even though these were immunized with higher doses and challenged with lower doses of SPZ. Although there was a significant delay in the onset of parasitaemia of 1 day of high-dose SPZ-SAS­(CL307)-immunized mice compared to RPMI controls (*p* < 0.001; Figure 4C), there was no difference in parasitic liver loads after challenge (*p* = 0.138; Figure 4F), again highlighting the importance for SPZ viability in conferring protection to malaria challenge.

### SGE-MMP­(CL307) Immunization Does Not Induce Protection

To exclude the effects of adjuvanted mosquito salivary gland extract (SGE) surrounding the SPZ in inducing protection, we included an SGE-MMP­(CL307) control. However, none of 9 mice immunized with matched amounts of adjuvanted SGE-MMP­(CL307) were protected or even delayed compared to RPMI controls, proving that the adjuvanted SGE had no effect on the protection (Figure 5A). Additionally, there is no significant difference in postchallenge liver load compared to either SGE or RPMI (*p* = 0.094 and *p* = 0.931, respectively, Figure 5B), so no aspecific immune effects were caused by the SGE.

**Figure 5:**
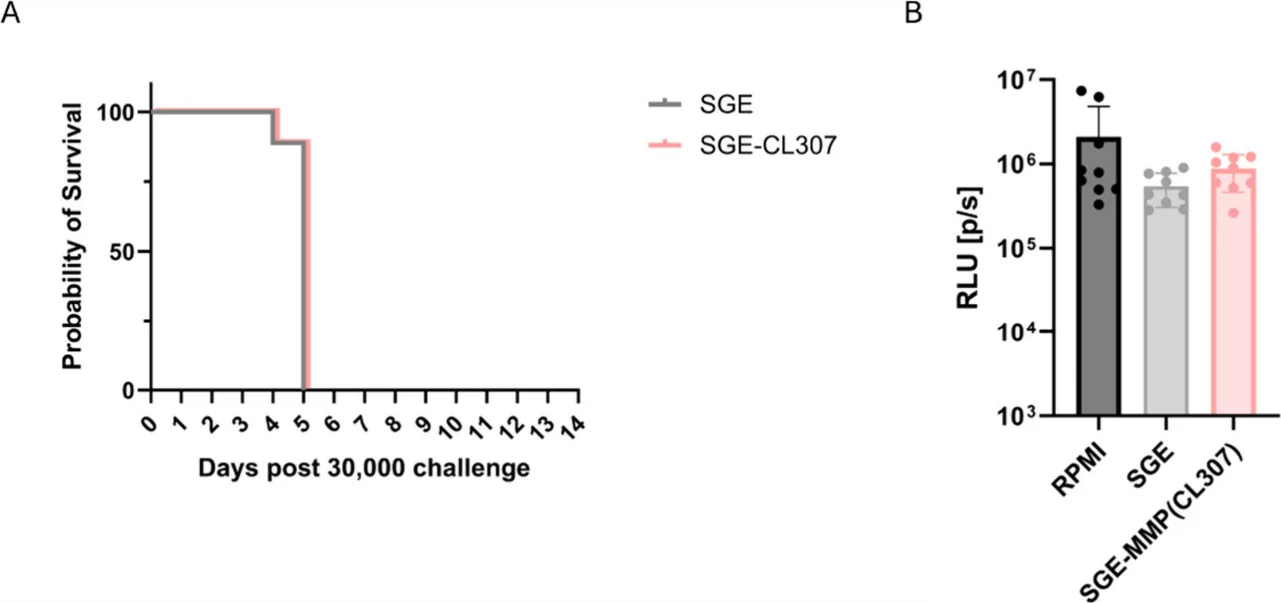
SGE-MMP­(CL307) immunization alone does not confer any protection: (A) Kaplan–Meier survival curves of adjuvanted SGE (light red) and control unadjuvanted SGE (gray) groups showing exact overlay of the curves. (B) Liver loads in RLU for RPMI (black), SGE (gray), and SGE-MMP­(CL307) (light red) groups after challenge with 30K SPZ. The immunization dose is matched to the amount of salivary gland material of the 5K unadjuvanted SPZ dose. SGE-MMP­(CL307) is compared with the two other groups using an independent sample Mann–Whitney *U* test.

## Discussion

By exploiting mitochondrial membrane potential uptake, we were able to load live cells with immunogenic moieties (adjuvants), with the goal of orchestrating the immune response. We generated adjuvanted SPZ (SPZ-MMP­(CL307)) that remained as infective and protective in C57BL/6 mice as unadjuvanted SPZ controls. We thus provide proof-of-concept data which demonstrate that chemical augmentation of live cells, such as SPZ, is possible while preserving the key viability features and consequently the possibility to deliver the adjuvant augmentation at the natural site of migration of the live carrier cell.

For malaria, this work adds to prior efforts to use live SPZ as immunogens. Because viability of SPZ is critical for protection,^3,4^ we have now focused our efforts on designing a platform which could allow for the delivery of adjuvant augmentation in live cells. The rodent model described in the current manuscript provides a screening platform for the most promising immunostimulatory pathways for SPZ.^26^ Beyond malaria, this method can have a broader application, as the generic concept can be applied to any live cell vaccine.

Although CL307 is a potent TLR7 agonist capable of activating murine immune responses, as previously demonstrated with SPZ-SAS­(CL307) and in early malaria infection,^16,27^ TLR7 adjuvanting in our hands did not significantly increase the protective efficacy of SPZ-MMP­(CL307) compared to unadjuvanted SPZ. We hypothesize that the CL307 adjuvant may not have reached a critical concentration at its target site in the liver. We thus argue that fine-tuning the mitochondrial loading to increase the amount of agonist per SPZ may hold potential to increase protective efficacy. Elucidating the mechanisms underlying the cytosolic uptake observed in Figure 1C could also help improve SPZ loading rates. A parallel strategy may be to use other TLR agonists that may be more potent at lower doses.^28^ At the same time, the loading of adjuvant cocktails holding different chemical compositions could be exploited to target multiple TLRs simultaneously, which is known to significantly enhance immunological effects.^29^ Moreover, different strategies than TLR agonist adjuvanting can be explored, such as different stimulants, chemoattractants, or antagonists, which might target a broader range of different cell types. Particularly those immune modulators that are specifically designed to activate innate immune pathways in hepatocytes may be worth exploring.^30–32^ MMP thus provides a platform that opens the way for innovative future research lines that explore careful orchestration of whole cell vaccine immune responses.

Augmentation of the immune response to whole SPZ has been tried before in multiple studies using adjuvant 7DW8-5 ^33–35^ or coadministration with either OX40 monoclonal antibody^36^ or a PD1 checkpoint inhibitor.^37^ All strategies led to an increase in IFNγ+ CD8+ T cells which translated into enhanced protective efficacy. This is why we explored the use of TLR7 agonists as adjuvants. TLR7 agonists are known to increase both the magnitude and polyfunctionality of CD8 T cells in viral models,^17^ suggesting the addition of TLR7 to SPZ could also induce increased CD8 T cell responses and thus protection. However, we have not directly investigated the effects of the loading of the TLR7 agonist on CD8+ T-cell responses targeting SPZ in the liver *in vivo* but rather investigated their protective potential using a rodent malaria challenge. An advantage of the small-molecule TLR7 agonist is that it is chemically amenable for our “Trojan horse” concept, allowing for targeted adjuvanting instead of systemic coadministration. The lack of protective efficacy *in vivo* in our current study could be related to the relatively low amount of the TLR7 agonist loaded in the SPZ, the lack of TLR7 target receptors inside hepatocytes,^32^ or inappropriateness of TLR7 agonists inducing IFN-I signaling for adjuvanting SPZ altogether.^38^ Future research is directed to untangle the roles of these individual components to understand the best path forward for chemical adjuvantation of SPZ.

To facilitate rapid evaluation of an innovative chemical adjuvanting modality, we opted for a short-term evaluation of protective efficacy *in vivo*. Whether this also translates to long-term memory and protection remains to be investigated. Given our findings, we think that further refinements in the adjuvanted-SPZ design are warranted before moving along in the translational track. Previously, when this technology was evaluated *in vitro*, an increase in CD8 T cell activation was observed,^21^ but we have not verified this *in vivo*. Given the theoretical potential of our technology to enhance T-cell responses and consequently induce more robust and longer memory responses, we consider that pursuit of further refinements and validation of its potential in rodent models is a valid forward strategy.

Within the specialized drug delivery platform field, this work provides a proof of concept of compound loading into live carrier cells without disrupting their migration pattern or other intracellular processes. Loading natural cells with therapeutics could represent the next advancement beyond biomimetic nanocarriers, which already offer improved cytocompatibility, target selectivity, and reduced immune activation.^39,40^ Unlike these approaches that engineer synthetic carriers to mimic cellular properties, the MMP-based loading strategy enables direct drug incorporation into living cells. This could yield an ideal delivery system (nontoxic, biodegradable, and inherently biocompatible) capable of precise target engagement without immune recognition as foreign.^41^

MMP is a known target for cellular fluorescent staining^42,43^ and loading of cells with mitochondria-targeting drugs.^44,45^ Compared to other cells, SPZs are very fragile unicellular organisms, holding but a single mitochondrion. Hence, it is highly likely that exploitation of MMP to load immunostimulatory molecules can also be used to strengthen cell therapies for other indications, including the many currently under clinical evaluation.^46^ For example, BCG vaccination or cell-mediated cancer therapies could benefit from this approach by converting cells that home to specific anatomical sites into live vehicles for localized adjuvant or drug delivery.^47,48^ Interestingly, these applications do not stop at single-cell organisms. We have, for example, also been able to use MMP directed uptake to load multicellular parasites (cercariae) with a dye and follow their penetration across host cell membranes.^49^

## Conclusions

Our study revealed valuable insights into the chemical augmentation of whole sporozoite vaccines. Although SPZ-SAS­(CL307) was loaded with more adjuvant than SPZ-MMP­(CL307), SPZ-SAS­(CL307) could not confer protective efficacy. SPZ-MMP­(CL307), however, was as protective as nonadjuvanted SPZ controls. We thus demonstrated that MMP provides a platform for the augmentation of live cells, opening avenues for screening of adjuvant moieties beyond CL307 for the augmentation of SPZ and applying MMP cell loading more broadly.
